# Application of Artificial Intelligence in Cone-Beam Computed Tomography for Airway Analysis: A Narrative Review

**DOI:** 10.3390/diagnostics14171917

**Published:** 2024-08-30

**Authors:** Izzati Nabilah Ismail, Pram Kumar Subramaniam, Khairul Bariah Chi Adam, Ahmad Badruddin Ghazali

**Affiliations:** 1 Oral and Maxillofacial Surgery Unit, Department of Oral and Maxillofacial Surgery and Oral Diagnosis, Kulliyyah of Dentistry, International Islamic University, Kuantan 25200, Malaysia; maxfacor@iium.edu.my (P.K.S.); bariah@iium.edu.my (K.B.C.A.); 2 Oral Radiology Unit, Department of Oral and Maxillofacial Surgery and Oral Diagnosis, Kulliyyah of Dentistry, International Islamic University, Kuantan 25200, Malaysia; badruddinghazali@iium.edu.my

**Keywords:** cone-beam computed tomography, CBCT, artificial intelligence, AI, airway analysis

## Abstract

Cone-beam computed tomography (CBCT) has emerged as a promising tool for the analysis of the upper airway, leveraging on its ability to provide three-dimensional information, minimal radiation exposure, affordability, and widespread accessibility. The integration of artificial intelligence (AI) in CBCT for airway analysis has shown improvements in the accuracy and efficiency of diagnosing and managing airway-related conditions. This review aims to explore the current applications of AI in CBCT for airway analysis, highlighting its components and processes, applications, benefits, challenges, and potential future directions. A comprehensive literature review was conducted, focusing on studies published in the last decade that discuss AI applications in CBCT airway analysis. Many studies reported the significant improvement in segmentation and measurement of airway volumes from CBCT using AI, thereby facilitating accurate diagnosis of airway-related conditions. In addition, these AI models demonstrated high accuracy and consistency in their application for airway analysis through automated segmentation tasks, volume measurement, and 3D reconstruction, which enhanced the diagnostic accuracy and allowed predictive treatment outcomes. Despite these advancements, challenges remain in the integration of AI into clinical workflows. Furthermore, variability in AI performance across different populations and imaging settings necessitates further validation studies. Continued research and development are essential to overcome current challenges and fully realize the potential of AI in airway analysis.

## 1. Introduction

Cone-beam computed tomography (CBCT) represents a promising technological advancement in imaging that can be used to explore the intricate structures of the craniofacial region including the upper airway anatomy and physiology. Recent advancements in data analytics and computing have facilitated the rise of artificial intelligence (AI) in medical imaging, enabling clinicians to quickly and accurately analyze CBCT scans [1].

Considered an invaluable tool in various dental and maxillofacial applications, CBCT provides detailed three-dimensional (3D) imaging with lower radiation exposure compared to conventional CT scans, allowing for a more efficient and precise assessment of bony structures in the maxillofacial region [2]. The high resolution of these images delineates the details of bony changes, allowing for the detection of trabecular bone alterations, bone erosion, and other periosteal bony reactions in disorders such as osteonecrosis, temporomandibular joint disorders, and alveolar bone defects. This aids in accurate diagnosis and staging, as well as monitoring disease progression, as CBCT can visualize subtle changes in bone density and architecture [2,3]. CBCT also provides critical information in assessing skeletal discrepancies, bone quality and volume, and the proximity of vital structures, enabling more accurate planning of surgical movements in orthognathic surgery [4] and the placement of dental implants [5], thereby reducing the risk of complications and ensuring better outcomes [6].

The ability of CBCT to analyze airways lies in the detailed assessment of dental and craniofacial morphology. It has been understood that a certain craniofacial phenotype will lead to an increased risk of obstructive airway disorders [7]. The upper airway is a hollow conduit made up of the craniofacial skeleton surrounded by the nasal, oral, and pharyngeal soft tissues. These structures can be clearly delineated in CBCT through several methods. Cross-sectional views of the airway in sagittal, axial, and transverse planes help identify the location and extent of airway constrictions. The segmentation of these images can produce three-dimensional and volumetric data. This enables accurate measurement of the volume of the airway, which is important in diagnosing and especially in assessing the treatment success of upper airway disorders.

The segmentation of CBCT images involves pre-processing to reduce noise and minimize artefacts while also defining the ROI that includes the airway. These data are then transferred to a specialized software such as Mimics v19.0 (Materialise, Leuven, Belgium), Dolphin Imaging Software (Patterson Dental Holdings, Inc., Chatsworth, CA, USA), or 3D Slicer (http://www.slicer.org, accessed on 22 August 2024) to process the segmentation. Various methods of segmentation process include manual, semi-automated, and automated segmentation. Because of the complexity of the airway anatomical structures, especially at the nasopharyngeal level where curvatures of the turbinates and soft palate make it challenging, effortful, and time-intensive to perform manual segmentation, it requires slice-by-slice segmentation of the datasets prior to performing the cutting plane passing [8,9]. This is where AI can be applied to ensure clinicians have a more efficient work load. 

There has been a surge of studies in the literature attempting to apply AI in their routine CBCT analysis for various indications such as growth and age prediction [10], detection of dental periodontal pathologies [11,12], and orthodontic diagnosis and virtual planning [13]. Despite the growing usage of AI in CBCT, the application in CBCT airway analysis are still lacking. Recent reviews claim that despite the accuracy and efficiency of automatic segmentation in airway analysis, there is insufficient evidence to support its clinical implementation at present [14,15]. Hence, the objective of this narrative review is to offer current knowledge and usage of AI-assisted CBCT upper airway analysis in diagnosing upper airway-related issues. In addition, this review also aims to highlight the components and processes of AI, its benefits, challenges, and potential future directions in CBCT airway analysis to equip surgeons with the fundamental knowledge to appreciate the opportunities presented by AI.

## 2. Background

Initially, the more versatile conventional computed tomography (CT) was used for 3D imaging in various medical applications due to its ability to provide highly detailed images, especially in soft tissues, as well as its potential for producing dynamic images, such as in the dynamic multi-detector CT, which allowed for the assessment of airway dynamics in functional airway disorders. However, this functionality is uncalled for in dental applications because it involves high radiation exposure, is costly, has limited availability in dental settings, and is often uncomfortable for patients [16].

This led to the development of CBCT technology, which became the preferred option in dental and maxillofacial application. Since then, the technology has been adopted by the industry and dental community, with more than 270 models having been developed and marketed worldwide [17]. It uses a rotating cone-shaped x-ray beam and a two-dimensional digital array detector placed in front of the patient’s head to collect volumetric data. During a single 360-degree scan, the x-ray source and detector move around the head in synchronization, acquiring basis projection images at intervals, which together form the projection dataset to provide multiplanar and three-dimensional reconstruction images [18]. 

CBCT is able to reduce up to 98% of its ionizing radiation doses compared to conventional CT [19], making it safer for patients, especially for repeated use in dental settings. It provides high-resolution 3D images for a smaller, focused field of view (FOV), which can be specifically tailored in dental and maxillofacial regions of interest (ROIs) for applications such as implant planning, endodontics, and evaluation of maxillomandibular anatomy, including upper airway analysis [20]. CBCT is more efficient and comfortable for dental settings as the machines are much smaller and have faster scanning processes than traditional CT scanners. In addition, the ability to manipulate the digital images allows for the processing of 2D and 3D images to produce a volumetric reconstruction and quantification, which are vital in airway analysis. 

With the increase in use of CBCT, multiple guidelines have been developed by various institutions to actively develop a framework for the use of CBCT [21,22,23]. Apart from using it for dental-related procedures, CBCT is actively researched and used for airway assessment [24]. According to the above guidelines, to ensure pharyngeal airway is included in the CBCT imaging, a large field of view (FOV) is required, for example, 15 × 15 cm, to ensure image analysis can be performed for accurate diagnosis of airway-related issues [25]. 

Previously, a two-dimensional approach using lateral cephalometry was used to examine the upper airway. However, this radiographic imaging has its limitations in providing a three-dimensional view of this complex structure [26], as it lacks cross-sectional and volumetric data, which are essential to reach an airway diagnosis. Meanwhile, CT or magnetic resonance imaging (MRI) scans can be too extravagant of an investigation to screen early or mild upper airway issues. Therefore, CBCT proves to be on par with the gold standard for upper airway assessment in measuring upper airway volume and identifying obstructive sites [1,27,28].

Recent advancements in AI have further enhanced the capabilities of CBCT, particularly in the segmentation of anatomical structures in CBCT images, a critical task for diagnosis and treatment planning of various dental and maxillofacial procedures. AI can be applied on CBCT for tooth segmentation, which can be beneficial in diagnosis of lesions in CBCT, and assist in endodontic treatment, orthodontic treatment planning, 3D-guided implant surgery, and auto-transplantation of teeth [29,30,31]. In addition, AI has the ability to enhance the quality of CBCT images by minimizing noise and reducing metal artifacts [32]. These advancements are particularly beneficial in clinical settings, enabling more precise diagnoses, better treatment planning, and improved patient outcomes across various dental and maxillofacial applications. 

**Figure 1:**
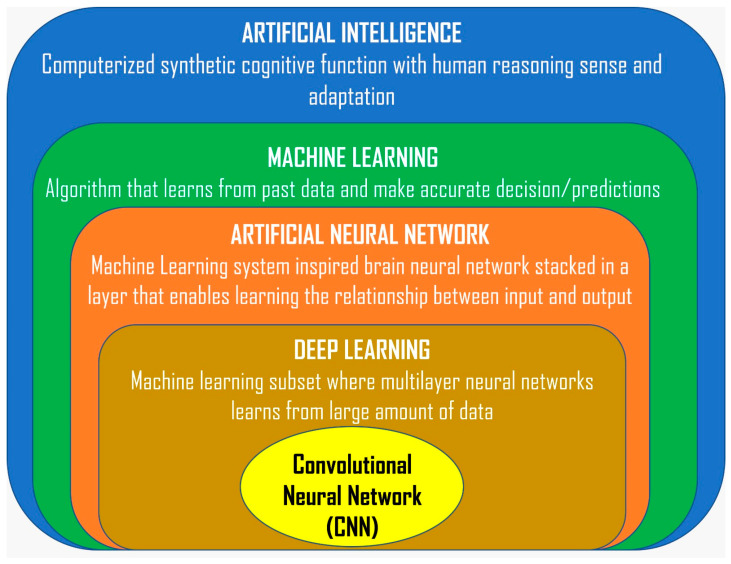
Overview of artificial intelligence and its elements.

Artificial intelligence is a computerized synthetic human cognitive process by means of machine system or computer program [1]. AI is currently gaining loads of recognition, and its use in the field of medicine is increasingly relevant, namely in radiology, specifically for analysis and interpretation of continuous scanned imaging such as MRI, CT, ultrasound, and CBCT. Machine learning (ML), deep learning (DL), and artificial neural networks (ANNs) are subsets of AI [33,34,35]. Figure 1 shows the overview of AI and its elements. 

ANNs and DL are fully automated systems that are currently preferred, as they can process high volumes of data in the shortest time possible. The term “neural” in ANN is coined due to the resemblance of neurons in brain on signaling function [36]. In simple term, neural networks work on principle of a nodal layer, which usually has an input layer, one or more hidden layers, and an output layer [37]. Each node is essentially an artificial neuron connected to the next node, and it has to hold a specific weight and threshold value. Once a value is above a node’s threshold value, the node is activated, allowing its data to transfer onwards to the network’s next layer. If the value is below the threshold value, no data pass along. Using a learning algorithm, this is achievable to be applied on CBCT data to allow automated segmentation and measurement [38].

Training data are fed into the neural network system and help to improve the system’s accuracy over time. A fine-tune learning algorithm allows rapid categorization and data congregation, which enables ANNs to recognize complex patterns in data to generate better outcomes and even produce predictions. Deep learning in ANNs alludes to the depth of node layers present within the network. An ANN is used in CBCT scans to analyze the airway by means of a convolutional neural network (CNN) that adopts an encoder-decoder or squeeze-and-excitation format structure. The airway space determination is identified by threshold-based information, semantic information, and geodesic and spatial information distinction. 

A convolutional neural network is designed for processing structured grids, namely images. A CNN can be thought of as an automatic feature extractor from the image. A CNN’s key components include the convolutional layer, pooling layer, activation function, and fully connected layer [39]. The convolutional layer applies convolutional operations to input images using filters to detect features, edges, textures, and complex patterns and preserve spatial relationships between pixels [40]. The pooling layer down-samples the spatial dimension of input, thus reducing computational complexity and numbers of parameters within the network [39]. The activation function, namely a non-linear type activation function, introduces non-linearity to the model, making it learn complex relationships in the data. Finally, the fully connected layer is responsible for making predictions based upon high-level features learned from the previous layer. They connect every neuron in one layer to every neuron in the next layer.

## 3. Application of AI in CBCT in Airway Analysis

**Figure 2:**
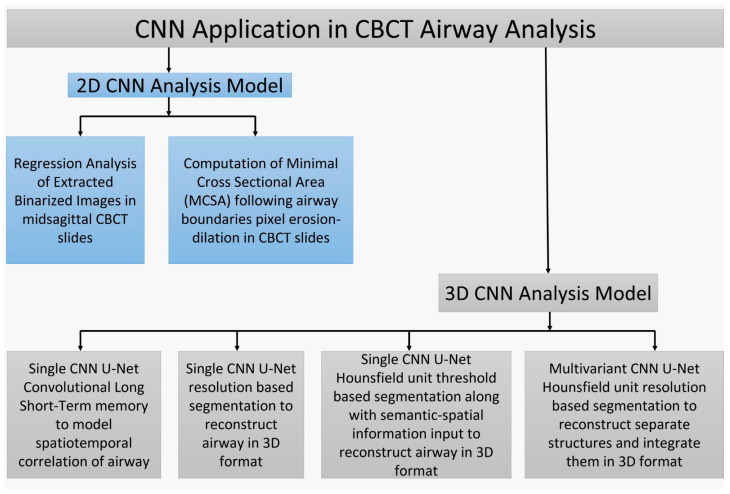
Summary of the CNN application in CBCT airway analysis.

Figure 2 summarizes the applications of a CNN-based deep learning algorithm used in upper airway analysis of CBCT scans. Table 1 summarizes the conceptual contributions of the reviewed articles on the application of AI using CBCT images for airway analysis, including their beneficial results.

### 3.1. 2D CNN Regression-Based Model

In this model, firstly, five location points are predicted on the mid-sagittal plane of CBCT by the 2D CNN for airway segmentation. The image is binarized, meaning the image is converted into two color format black and white. The same image is also hole-filled via closed operation mode. The binarized image is subtracted from the hole-filled image, leaving only the airway space image. The learning algorithm will automatically subdivide the airway image using the five-location marking point, and the ¼ and ¾ borders are connected. This airway space volume can be measured accordingly [41]. 

### 3.2. 3D CNN U-Net Resolution-Based Model

This model is trained with binary cross entropy loss formulae, and algorithm learning is conducted with DICOM images [14]. Automatic airway segmentation is performed by first converting the full CBCT scan to a fixed size at lower resolution. The scan is uploaded to a U-Net already trained to segment the low-resolution scan. Next, the low-resolution approximation is used to select several full-resolution patches. The patch segmentations are combined to form a final segmentation map, which becomes the final 3D reconstructed pharyngeal airway space in STL format. 

### 3.3. 3D CNN U-Net Threshold Value-Based Pipeline Model

Initially, the CBCT scan images are pre-processed first. The image segments irrelevant to the airway region are cropped off in the axial plane to reduce computational load. Additionally, scan segments containing brain structures are also cropped off in the sagittal plan. However, CBCT image size reduction is avoided, as it will cause airway volume measurement problems. The U-Net model is an encoder-decoder algorithm that helps the program to capture semantic information and recover spatial information of the airway to create output. The output is viewed as extracted airway volumetric image slice layers that are stacked to form the airway 3D model. Semantic segmentation is performed by categorizing each pixel as airway or as background, while spatial information is the imaging modality data to differentiate two adjacent structures as being distinctive from each other [15].

### 3.4. Multivariate 3D CNN U-Net Resolution-Based Model 

CBCT in Digital Imaging and Communications in Medicine (DICOM^®^) format is uploaded into a cloud-based online platform that incorporates multiple stacks of various 3D U-Net CNN systems. The CBCT image data are pre-processed to form a lower-resolution image. These lower-resolution images become the input for the neural network. The CNN outlines the convolution layers, coding contraction path, and symmetric decoding expansion path that forms a U-shaped architecture. The platform allows segmentation of distinguishable structure such as mandible, dentition, maxillofacial complex, pharyngeal airway, maxillary sinus, and mandibular canal. Each segmentation structure initiates a unique AI pipeline, and the output from these pipelines is the automatic transformation of individual 3D models in STL format that are combined into a single segmentation map that allows visualization of each class of volume voxel. The integrated segment objects can also be exported in an STL file [42].

### 3.5. CNN U-Net Convolutional Long Short-Term Memory-Based Model 

This model is a CNN U-Net encoder-decoder based on the Tiramisu model that is further fortified by a squeeze-and-excitation block, which allows for suitable calibration of the most important feature and convolutional long short-term memory to model spatiotemporal correlation between regions of interest in the consecutive CBCT slides. Some key features in this model include the fact that the convolutional long short-term memory is used to narrow the junction layer of the neural network to exploit the spatial axial correlation of consecutive scan slices instead of processing each CBCT scan individually. The down-sampling and up-sampling network path, with the addition of squeeze-and-excitation layers, improves the representational power of the model and feature interpretation at a later stage [43].

### 3.6. 2D CNN Minimal Cross-Sectional Area (MCSA) Localization Model

Initial segmentation images either by manual or AI-generated type are acquired first as a dataset. A non-linear mathematical morphological operation comprising erosion and dilation is used to process the segmentation images. The inner boundary is extracted using pixel erosion to create a contraction of the original image while the outer boundary is extracted using the dilation processing method. An exclusive OR operation is conducted on the contraction result and target image as well as dilation result and target image to obtain a difference set. The MCSA is automatically determined via computation once the erosion-dilation application extracts the binary image boundaries. This is performed by calculation of the sum of pixel numbers in each to measure the width of the upper airway at various levels. The narrowest slice with the minimum pixel number is selected and subjected to originating the coordinate system. The narrowest heights will then be recorded [44].

## 4. Benefits of AI in CBCT Airway Analysis

The detailed 3D images produced by CBCT are invaluable for various diagnostic and treatment planning purposes and have garnered significant attention in the integration of AI in airway analysis for various airway conditions. This section explores the benefits of AI in enhancing the accuracy, efficiency, and overall effectiveness of CBCT airway analysis.

### 4.1. Enhanced Accuracy in Airway Volume Measurement

CBCT allows precise quantitative measurements of distances, angles, cross-sectional areas, surface areas, volumes, and shapes [24,46]. AI algorithms in CBCT, particularly those employing deep learning techniques, have shown remarkable accuracy in segmenting and analyzing airway structures. Traditional manual segmentation methods are time-consuming and prone to human error. AI can automate this process, reducing variability and improving the precision of measurements. Studies have demonstrated that AI-driven segmentation aligns closely with expert manual segmentation, offering a reliable alternative for clinicians [9,14,15,45]. Studies have also shown that even the 3D airway from CBCT data using a semi-assisted software program is accurate, reliable, and fast [43,47]. 

### 4.2. Improved Efficiency and Time Savings

One of the most significant advantages of AI in CBCT airway analysis is the reduction in time required for image processing. Traditional methods can be labor-intensive, often taking hours to complete. AI can perform these tasks in a fraction of the time, freeing up clinicians to focus on other critical aspects of patient care [48].

### 4.3. Enhanced Diagnostic Capabilities

AI is capable of detecting subtle anomalies and variations in airway anatomy that may be overlooked by the human eye. Machine learning models, trained on vast datasets, can identify patterns and correlations that might not be immediately apparent to clinicians. This capability enhances diagnostic accuracy and allows for earlier detection of airway-related conditions [41,49].

### 4.4. Standardization and Consistency

AI algorithms provide standardized analysis, ensuring consistency across different patients and clinical settings. This standardization is particularly beneficial in multicenter studies and collaborative research, where consistent data analysis is crucial for valid comparisons and conclusions. Additionally, the uniformity makes it possible to standardize patient care even when it is provided by untrained physicians [50]. A Mallampati assessment using CBCT was used to construct a model to predict airway obstruction. The program can interpret input data to match specified patterns and then generate a suggestion based on those patterns. If the Mallampati classification is “Class II”, for instance, mouth opening is “limited”, neck movement is “Limited”, and cervical spine stability is “Unstable”, the model will suggest to the anesthesiologist to employ airway adjuncts to improve stability throughout the treatment. Therefore, anesthesiologists can recieve personalized recommendations for managing airways by using this model of recommendations, which takes into account the unique features of each patient’s airway assessment [45,48].

### 4.5. Integration with Treatment Planning

AI can assist in creating personalized treatment plans by analyzing the CBCT airway data and predicting the outcomes of various interventions. This integration is particularly useful in orthodontics and surgical planning, where precise predictions can improve patient outcomes [13,51].

### 4.6. Continuous Learning and Improvement

AI systems continuously learn and improve from new data, enhancing their performance over time. As more CBCT airway data become available, AI algorithms become more robust and accurate, leading to ongoing improvements in clinical practice [52,53].

### 4.7. Safety and Privacy-Preserved Information

The AI in CBCT analysis may allow protection from cyber threats and patients’ information data leak via the privacy-preserved threat intelligent framework. There are a few research studies that propose a framework called the privacy-preserved threat intelligence framework to safeguard sensitive data and detect cyber threats within industrial Internet of Things environments [51,54,55].

The integration of AI in CBCT airway analysis offers numerous benefits, including enhanced accuracy, improved efficiency, better diagnostic capabilities, standardization, and integration with treatment planning. As AI technology continues to evolve, its applications in airway analysis are expected to expand, further improving patient care and clinical outcomes. Future research should focus on refining AI algorithms, expanding their training datasets, and exploring new applications in airway management and treatment.

## 5. Challenges and Limitations

### 5.1. Limited Data Availability

There are some limitations regarding the research and adoption of AI in clinical settings. The first point highlighted is the limited data availability and lacking comprehensiveness when developing AI algorithm. It is common to see the use of 1000 and more datasets in AI research, as having more comprehensive data available would be very much favourable [52]. However, a recent systematic review found that AI CBCT airway research is only using 40 to 300 CBCT datasets. Most of the research projects only utilized a single CBCT machine [1], while two articles [14,45] used two to three CBCT machines. The data training and test are usually coming from only one center with supervised learning methodology, as well as training complex AI models. In addition, current hardware can produce high carbon emissions, so a more sustainable alternative may be beneficial in the future [52].

Apart from these, the validity of any AI algorithm will depend on the CBCT data used to train it. Several factors influencing the quality of CBCT images, namely patient motion [56] and head positioning [57], artifacts [58], kV parameter [57], and inadequate FOV [56], may inadvertently affect the validity of the AI algorithm. Other technical factors such as electrical noise, scatter, beam hardening, as well as artifacts originating from dental materials are also known to reduce the quality of any CBCT images [59]. These factors lead to a limited number of CBCT datasets available for training the algorithm [38]. Additionally, AI models must be resilient enough to accommodate a range of airway anatomies across different backgrounds of patients [60]. Addressing these challenges involves a combination of better imaging practices, diverse and representative training datasets, and advanced methods for integrating clinical context into AI models.

### 5.2. Lack of Standard Methodological Framework

Lacking in methodological framework and standard during the development of AI algorithms in general is another pitfall to using AI in CBCT airway analysis. Medical data from healthcare providers such as hospitals and universities are considered sensitive to be shared with other researchers when compared to other types of data that can be used in artificial intelligence research [52]. Under the EU General Data Protection Regulation, medical data such as imaging data are owned explicitly by the patients, and a specific consent needs to be signed if the imaging data are to be used for AI algorithm development research, and this needs to be renewed for each version of the algorithm. Meanwhile, in the other parts of the world, the data are owned by the imaging centers, which makes the data sharing easier [61].

The goal of achieving the best value by ethical use of AI while enduring the lure of monetary gain from benefitting from the outcome of medical AI is very subjective. The development of AI from medical data requires not only those that are stored in the healthcare facility servers but also well-labeled radiology data created by medical professionals such as a radiologist and/or other medical and dental professionals, which will be used commercially once the algorithm is cleared by regulating bodies. This is another ethical issue that will require a continuous look, as the process is very dynamic and quickly evolving [62].

### 5.3. Selection and Interpretation Bias

The sampling process of these medical data can lead to selection bias by having too many healthy samples or too many diseased samples [11]. How the data were processed and measured and the result validation are frequently replicable in medical AI research. If the same data are used for both training and validation, it can lead to “data snooping bias”. Overfitting is a known problem in AI research, especially in medical data, as the availability of the training dataset may not represent the whole population. This is where the AI is more subjected to overly represented positive findings and over-interpret the disease [62]. Moreover, the radiological data are quite subjective, where the radiologists prefer terms such as “probably normal”, while mathematics and statistics used in AI algorithms prefer something absolute like “definitely normal” and “definitely abnormal”. Moreover, different medical professionals may not agree on the similar classification of disease when provided with similar medical data like imaging data. The ground truth labeling process with more than one medical professional is one of the ways to overcome this [62].

AI algorithms developed through supervised learning where the medical professionals labeled the ground truth may be subjected to human bias. This is because the labeling process may include personal preferences and human values that are used in training the AI algorithm [61]. The ethical considerations for development and use of AI are a dynamic process and will need to be looked again every once in a while.

To reduce the risk of bias in the development of an AI algorithm, it is suggested that patient characteristics be included in the training datasets during data collection and preparation. The training data should be carefully curated with a diverse patient cohort, and error evaluation should be performed for each cohort. Even after deployment and clinical authorization, a different type of bias may arise. A domain shift can occur when the patient cohort using the algorithm differs from the training data on which the algorithm was developed, potentially leading to inaccuracies. In such cases, new post-authorization data may be required [63].

There are also some checklists that can guide researchers, developers, and regulatory bodies to assess the risk of bias and ensure transparency from AI algorithms developed from medical or patient data [63]. PROBAST, which stands for Prediction model Risk Of Bias Assessment Tool, is a tool to assess the risk of bias and applicability of prediction model studies [64]. It includes detailed guidance on how to use the tool and interpret its findings, making it accessible for researchers, clinicians, and other stakeholders involved in the development or assessment of AI models. Other guidelines such as Minimum information about clinical artificial intelligence modelling: the MI-CLAIM checklist [65] and Checklist for Artificial Intelligence in Medical Imaging (CLAIM) [66] can provide a standardized framework for reporting essential information about the development, validation, and application of AI models in clinical settings, which will ultimately reduce bias in AI modeling.

### 5.4. Accessibility and Transparency Issues

Adequate computational infrastructure is necessary to run AI algorithms efficiently. Clinics without advanced computing resources may find it difficult to leverage AI technologies. Most current AI applications in CBCT offer only limited information, which only partially supports the complex decision-making required in clinical care. Additionally, issues regarding accountability and transparency persist, even for commercially available programs developed with AI [52].

Currently, there are no established guidelines for the minimum accuracy needed before it can be deployed in clinical practice. There is no concrete separation of allowable false positive and false negative threshold obtained by the AI algorithm for diagnostic purpose. Shift from a controlled, single institution research setup to real-world data may be challenging due to different patient populations and disease incidences that may be completely different from the carefully curated training dataset [67]. Rigorous validation of AI models is necessary to ensure their reliability and accuracy. Transparent reporting of validation processes and outcomes is essential for building confidence in AI tools.

All in all, the limited number of published research articles on this topic shows that it is still an important area of interest and the outcome from this topic is still in the early stages. More high-quality evidence with low risk of bias is needed to show the accuracy of the CBCT airway analysis using artificial intelligence [1].

## 6. Future Direction

### 6.1. Enhance Collaboration and Data Sharing

Moving forward, the potential advancements and development in integrating AI in CBCT airway analysis are promising. An effort to raise the performance of AI models to expert level should be employed to enhance the precision and accuracy in segmentation of CBCT airway analysis. Development of more sophisticated AI algorithms can be achieved through increased research efforts and collaboration among multiple centers to expand the datasets and variables [53]. Leveraging large datasets can enhance the precision and applicability of AI models.

### 6.2. Continuous Calibration and Validation

Furthermore, the dynamic learning process and constant unfolding of new datasets in AI necessitate continuous and meticulous review of datasets, alongside performance monitoring of algorithms. This ensures constant calibration and validation of AI capabilities while facilitating the implementation of airway analysis using CBCT in clinical practice. With AI automating CBCT to generate comprehensive airway analysis reports, the workload of radiologists and clinicians is expected to become more efficient.

### 6.3. Integration with Clinical Workflows

Development of more intuitive and user-friendly interfaces will make it easier for clinicians to interact with AI systems, reducing the learning curve and increasing adoption rates. Integrating AI with other technologies like wearable biosensors, 3D anthropometric scanners of the face and body, or even mobile applications alongside CBCT make screening for airway-related issues seamless and enhance the diagnostic ability [68]. On top of that, they can also be used over time at home for patients to provide continuous data that can be analyzed by AI to predict issues and track changes upon treatment. The incorporation of different AI tools into established clinical workflows ensures that they enhance rather than disrupt current practices.

### 6.4. Predictive Analytics

By employing serial CBCT images alongside AI algorithms, airway conditions can be predicted over the course of patients’ development while also evaluating treatment effectiveness post-intervention [69]. Using these datasets alongside a simulated virtual surgical plan, new predictive models can be developed to assist surgeons in real time during procedures to provide automated recognition and navigation of anatomical landmarks to enhance safety and precision during delicate manoeuvres of a certain surgical procedure. This, thereby, reduces the risk of complications.

### 6.5. Ethical and Regulatory Advancements

Lastly, development of standardized protocols and guidelines for the use of AI in CBCT airway analysis is essential to ensure responsible use of data, addressing concerns relating to bias, while also ensuring consistency and reliability of AI function. Conducting robust regulatory frameworks to validate the efficacy and safety of AI tools in CBCT airway analysis ensures that AI tools are used ethically, with a focus on patient privacy, consent, and unbiased analysis [61,62]. By focusing on these areas, the future of AI in CBCT airway analysis holds great potential for improving patient outcomes, enhancing diagnostic accuracy, and streamlining clinical workflows.

## 7. Conclusions

Airway analysis using CBCT scan images is more commonplace recently as either 2D or 3D analysis since the advent of various forms of AI systems that are rapidly moving onto full automation with little to no direct human intervention. The literature reviews in general show how with CBCT scans, AI systems are able to analyze the upper airway, namely the sino-nasal–pharyngeal region via a convolutional neural network (CNN), which is quickly becoming a mainstay AI modality due to its versatility and multi-variant processing capabilities through formulae and learning algorithms. Based on available recent studies, these deep learning neural network systems used on CBCT scans have shown impressive precision in segmentation and analysis of airway structure. This is particularly relevant, as the AI system is able to identify volumetric reduction or constriction in the airway accurately whilst reducing analysis time, increasing effective working time, integrating with medical-surgical treatment plans, and enabling constant learning as well as improvement of predictions. AI in CBCT analysis has better diagnostic capabilities now and is more consistent in its prediction or outcome, which is necessary for analysis output standardization.

However, AI algorithm’s preference of absolute or definitive output is not suited for medical imaging analysis due to potential medicolegal concern. Additional issues of need for adequate computation resources in order to integrate AI systems with CBCT while countermanding problems regarding accountability, patient confidentiality, and transparency code need to be considered. It is therefore essential that continuous calibration and validation by constant learning with new CBCT datasets and improving AI’s learning algorithm are conducted, which will improve airway analysis. At the same time, improving user-friendly AI interface systems that can integrate with clinical workflow as well as upgrading adjuvant visual and medical sensors is very useful from a clinician’s perspective. Still, ethical and regulatory protocols should be put in place, namely in the implementation of AI in CBCT airway analysis, thus ensuring data safety, minimizing unbiased analysis, and guaranteeing a patient’s privacy security. Hence, in summation, a continuous upgrade of CBCT images datasets alongside advancement of AI algorithms will yield better and more accurately reliable predictive models that are very necessary in diagnosis, treatment, and post-treatment phase assessments for supraglottic airway obstructive disorder, thus minimizing complications concurrently.

## Figures and Tables

**Table 1:** Summary of articles reviewed, highlighting the CBCT machine and technical specifications, number of CBCT data, type of AI application, image processing method and its software and hardware used, and the significant benefits of the AI models.

Author, Year, Location	CBCT Machine (Technical Specification)	No. of CBCT Data	AI Application	Image Processing Method—Software Used	AI Modelling—Software and/or Hardware Used	Beneficial Results
Shujaat et al., 2021, Belgium [14]	Promax 3D Max (Planmeca, Helsinki, Finland) (96 kV, 216 mAs, slice thickness: 0.6 mm, field of view: 230 × 260 mm^2^) Newtom VGi evo (Cefla, Imola, Italy) (110 kV, 15.3 mAs, slice thickness: 0.3, field of view: 240 × 190 mm^2^)	103	3D CNN U-Net resolution-based model	Segmentation of PAS volume limited by the nasal cavity, oral cavity, pharyngeal border until the limit of the scan either at 2nd, 3rd, or 4th cervical vertebrae. Delineation was based on resolution of Hounsfield unit to create mask in axial, sagittal, and coronal plane to convert to STL file format - Mimics software (version 22.0, Materialise N.V., Leuven, Belgium)	Own model Online customized user-interactive cloud-based platform (version 1.0, Toothflow, Relu, Inc., Leuven. Belgium)	- Accurate segmentation of pharyngeal airway space Time-efficient method
Sin et al., 2021, Turkey [15]	Newtom 3G (Quantitative Radiology srl, Verona, Italy) (120 kVp and 3–5 mA, 12in, 13.48 cm imaging field, axial slice thickness 0.3 mm, isotropic voxels)	306	3D CNN U-Net threshold value-based pipeline model	Semi-auto segmentation by determining thresholding values to isolate the anatomic region, then placement of seed regions for active contour model - Open software version 3.8 ITK-SNAP (http://www.itksnap.org/ accessed on 22 August 2024)	Own model - Based on MATLAB implementation of U-Net. Developed using NVIDIA^®^ GeForce^®^ RTX 2080 Ti GPU.	- High similarity rate between automatic and manual segmentation - Higher inter-correlation between AI and researcher, than intra-researcher - Error-free segmentation of pharyngeal airway volume
Park et al., 2021, South Korea [41]	PaX-i3D (Vatech Co., Hwaseong-si, South Korea) (105–114 KVP, 5.6–6.5 mA with 160 mm × 160 mm field of view, and 0.3 mm in voxel size)	315	2D CNN Regression-based models	5 coordinates predicted for airway segmentation in sagittal plane includes posterior palate, vomer, 1st, 2nd, or 3rd cervical vertebrae - Image processing using MATLAB 2020a (MathWorks, Natick, MA, USA)	Own model - Program developed based on MATLAB 2020a (MathWorks, Natick, MA, USA) Network training using NVIDIA Titan RTX GPU with CUDA (version 10.1)	- Good to excellent reliability - Fully automatic segmentation of the airway is possible by training via AI - High correlation between manual data and AI data
Nogueira-Reis et al., 2024, Belgium [42]	3D Accuitomo 170 (J. Morita, Kyoto, Japan) (90 kVp, 5 mA, 0.2–0.25 mm voxel size, FOV 17 × 12 cm, 14 × 10 cm, 10 × 10 cm) Newtom VGi evo (Cefla, Imola, Italy) (110 kB, 6–12 mA, 0.25–0.3 mm Voxel size, FOV 24 × 19 cm)	30	Multivariate 3D CNN U-Net resolution-based model	Six craniofacial structures, encompassing the maxillofacial complex bones, maxillary sinus, dentition, mandible, mandibular canal, and pharyngeal airway space, were segmented. Minor refinements were manually corrected. Refined segmentation served as reference for comparison. - Mimics Innovation Suite software (version 23.0, Materialise N.V., Leuven, Belgium	Own model - Training model done using cloud-based online platform known as Virtual Patient Creator (creator.relu.eu, Relu BV, Version March 2022)	- Accurate segmentation - Time-efficient through simultaneous segmentation Consistent CBCT-derived virtual patient
Leonardi et al., 2021, Italy [43]	iCAT Next Generation CBCT unit (Imaging Sciences International, Hatfield, Pa) (120 kVp; 48 mA; 0.3 mm voxel size; scan time, 26 s; field view of 17 cm in height × 23 cm in depth)	40	CNN U-Net Convolutional Long Short-Term Memory-based model	Landmarks and boundaries used include Nasion, second and third cervical vertebrae, porion, and orbitale. Segmentation mask of the sino-nasal cavity and pharyngeal subregion after the enhancement of boundaries performed by manually erasing the parts outsides the region of interest - Mimics software (version 20.0; Materialise, Leuven, Belgium)	Own model - Algorithm based on Tiramisu model - Data training done using Titan X Pascal GPU [NVIDIA Corporate, Santa Clara, Calif]	- High accuracy of automated and manual segmentation - Reduced method error
Chu et al., 2023, Hong Kong [44]	ProMax 3D Mid (Planmeca Oy, Helsinki, Finland) (96 kV, 216 mAs, slice thickness: 0.6 mm, field of view: 230 × 260 mm^2^)	201	2D CNN Minimal Cross-Sectional Area (MCSA) localization model	MCSA at three different levels using midsagittal plane: nasopharynx, retropalatal pharynx, retroglossal pharynx - Mimics v19.0 (Materialise, Leuven, Belgium)	Own model Model training based on Adam optimization algorithm and Pytorch framework using Intel i7-8700 CPU, 32GB RAM and a single Nvidia RTX 2080 Ti GPU with 12G VRAM (Jumbo computer supplies, Hong Kong, China)	- High precision in all analysed models - Consistent MCSA localisation More efficient AI processing of airway segmentation and MCSA localisation.
Orhan et al., 2022, Denmark [45]	Pax-i3D Smart PHT-30LFO0 (Vatech, Gyeonggi-do, South Korea) Carestream Health CS 8100 3D (Kodak, Rochester, NY, USA), Orthophos XG 3D (Sirona, Germany) isotropic voxels which differ between 0.1 and 0.2 mm^3^	200	3D CNN U-Net resolution-based model	Automatic segmentation focusing on external surface of bones, teeth, and airways: - InVivo 5.1.2 (Anatomage Inc., San Jose, CA, USA) Series of trials to choose best training configuration. Generated STL files used for volumetric pharyngeal airway measurements - Materialise 3-Matic Version 15.0 (Materialise N.V, Leuven Belgium)	Diagnocat (DGNCT LLC, Miami, FL, USA) Training using NVIDIA GeForce RTX A100 GPU	- Accurate automatic detection of OSA Increase diagnostic accuracy

## Data Availability

Not applicable.

## Abbreviations

3D	Three-dimensional
CBCT	Cone-beam computed tomography
AI	Artificial intelligence
FOV	Field of view
ROI	Region of interest
MRI	Magnetic resonance imaging
ML	Machine learning
DL	Deep learning
ANN	Artificial neural network
CNN	Convolutional neural network
MCSA	Minimal cross-sectional area
PAS	Pharyngeal airway space
STL	Stereolithography
DICOM	Digital Imaging and Communications in Medicine
PROBAST	Prediction model Risk Of Bias Assessment Tool
MI-CLAIM	Minimum information about clinical artificial intelligence modelling
CLAIM	Checklist for Artificial Intelligence in Medical Imaging
